# Mechanochemical Activation Effect on Technogenic Iron Oxide Reduction Kinetics

**DOI:** 10.3390/ma15010320

**Published:** 2022-01-03

**Authors:** Oleg Sheshukov, Mikhail Mikheenkov, Larisa Vedmid, Denis Egiazaryan

**Affiliations:** 1 Institute of Metallurgy of the Ural Branch of Russian Academy of Sciences, 101 Amundsen Str., 620016 Ekaterinburg, Russia; o.j.sheshukov@urfu.ru (O.S.); Silast@mail.ru (M.M.); avari@mail.ru (D.E.); 2 Federal State Autonomous Educational Institution of Higher Education «Ural Federal University Named after the First President of Russia B. N. Yeltsin», Mira Str. 19, 620002 Ekaterinburg, Russia

**Keywords:** scale, iron oxides, recovery, diffusion, mechanochemical activation, defects of the crystal lattice, kinetics

## Abstract

Understanding the reaction kinetics of iron oxide reduction by carbon is a key task of the theory of metallurgical processes. One of the understudied features of the reaction kinetics of iron oxide solid-phase reduction by carbon is the discrepancy between the reacting substances’ small contact area and the process’s high rate. A convincing theoretical and experimental explanation of this effect has not yet been obtained. The data obtained earlier show that an increase in the scale of the briquetting pressure from 0 to 300 MPa increases the degree of its metallization during heating two-fold, and the metallization temperature decreases by more than 40 °C. Therefore, it was assumed that these effects during heating are a consequence of the mechanochemical activation (MCA) of iron oxides in the scale during its pressing. This paper presents the results of experimental studies on the influence of two types of scale MCA (grinding and pressing) on iron oxide reduction. The study of the MCA effect on the reaction kinetics of scale iron oxide reduction by carbon is a promising way to assess the criteria for scale phase composition changes under external factors. The presented results indicate a decrease in the amount of trivalent iron oxide (Fe_2_O_3_) after the MCA and an increase in the amount of one-and-a-half oxide (Fe_3_O_4_) and bivalent iron oxide (FeO). The obtained experimental data show that the initial stage of iron oxide reduction, consisting in the transition from higher iron oxides to lower ones, is possible at room temperature without carbon presence.

## 1. Introduction

Throughout the centuries-old practice of iron ore reduction, solid carbon in the form of wood, coal and coke has become the most widespread reductant. When reducing iron oxides with carbon, both initial reagents and some reaction products are in a solid state. Therefore, the first attempts to scientifically substantiate this process, which arose in the 19th–20th centuries, focused on describing the reagents’ solid-phase interaction. At the same time, when describing the solid-phase iron reduction, a discrepancy between the reacting substances’ small contact area and the high rate of iron reduction was noted. To explain this effect, a significant number of theories have appeared, the founders of which are G. Tamman, G. Heavenshi, K. Tubandt, J. O. Endstrom, and others. However, convincing experimental data and theoretical justification of the high rate of solid-phase reduction of iron oxides by carbon have not yet been obtained. Most researchers into the solid-phase model of iron oxide and carbon interaction explain the reduction’s high rate by the presence of defects in the iron oxides’ crystal lattices, through which the ions of reacting substances are diffused. In recent years, a large number of works have appeared on the MCA effect on the kinetics of solid-phase reactions [1], in which an increase in the reaction rate is noted, and this effect is explained similarly, by the appearance of a large number of defects in the reacting substances’ crystal lattices.

It should be noted that the solid-phase interaction simulation of iron and carbon oxides was carried out during heating, under conditions close to industrial processes [2], without taking into account the preparation of raw materials. As a result, the industry has developed a classic scheme for preparing ore raw materials for blast furnace smelting by manufacturing pellets and agglomerate. Such preparation is carried out at atmospheric pressure without paying attention to the grinding degree of the raw materials. It is obvious that such a preparation cannot provide the proper area and contact strength of the reacting substances which contribute to the solid-phase reaction acceleration. Briquetting of metallurgical wastes together with blast furnace slag has been proposed for various forms of iron oxide reduction, up to metallic iron production [3].

In [4], the carbothermic reduction of rolled scale in an argon atmosphere was investigated by thermal analysis. It is established that the reduction proceeds in the sequence Fe_3_O_4_-FeO-Fe_3_C-α-Fe. In [5], the MCA influence (the briquetting pressure of the initial reagents) on the reduction in kinetics of scale of iron oxides during heating is considered. It was found that the reduction of iron oxides in scale proceeds in a solid-phase manner in three modes: diffusion-free, intermediate and diffusion. In the diffusion-free mode, the rate of heterogeneous reaction between iron oxides and carbon is limited by the contact area of the reacting substances and the contact’s pushing pressure. In this mode, the pushing pressure has a significant effect on the reduction reaction’s rate. In the diffusion-free mode, with the scale briquetting pressure increasing from 0 to 300 MPa, the metallization degree increases from 34.6 to 71.9%, and the metallization temperature shifts down from 1021.7 to 989.8 °C. In the intermediate mode, the diffusion processes start to affect the iron oxides’ reduction, but the briquetting pressure effect on the reduction kinetics is partially preserved. In the intermediate mode, with an increase in the scale briquetting pressure from 0 to 300 MPa, the metallization degree increases from 22.8 to 67.2%, while the metallization temperature in the entire range of briquetting pressures shifts from 930.7 to 947.6 °C. In the diffusion mode, due to the presence of a liquid silicate phase, only the diffusion mechanism works and the briquetting pressure has practically no effect on the reduction rates. In the diffusion mode, with an increase in the scale briquetting pressure from 0 to 300 MPa, the metallization degree practically does not change and is in the range of 58.2–73.6%, and the metallization temperature shifts from 883.7 to 911.7 °C.

In [5], only one of the MCA methods was considered (pressing). However, preliminary grinding is important for scale secondary processing. In the literature there is a significant amount of work on another MCA method (grinding to high specific surfaces of the oxide material). It was shown in [6] that mechanical treatment of Fe and α-Fe_2_O_3_ mixture in a planetary centrifugal mill leads to the formation of a nanocrystalline wustite (FeO) of nonequilibrium composition. This forms a nanoscale Fe/Fe_3_O_4_ composite during subsequent thermal decomposition in vacuum at 200 °C. The MCA effect on the monophase powder of α-Fe_2_O_3_ by grinding it in a roller-ring and planetary mills was studied in [7]. It is shown that during mechanical activation, the α-Fe_2_O_3_ phase composition remains unchanged, with only a slight decrease in the coherent scattering regions and an increase in the micro-deformations’ magnitude. According to the authors, the increase in the α-Fe_2_O_3_ micro-deformations’ magnitude during mechanical activation is associated with an increase in crystal structure defects. In [8], the effect of MCA by grinding in a high-energy planetary ball mill on the properties and structure of monophase α-Fe_2_O_3_ and α-Fe_2_O_3_ grounded together with graphite was studied. It is established according to X-ray phase analysis that during monophase α-Fe_2_O_3_ grinding, with an increase in the grinding duration the size of the coherent scattering region decreases and amorphization of the hematite (Fe_2_O_3_) crystal structure occurs. The last is reflected in the interplane distances’ diffraction maxima broadening, and in a decrease in their intensity. During α-Fe_2_O_3_ and graphite grounding, the number of Fe^3+^ ions decreases, the number of Fe^2+^ ions increases, and this leads to Fe_3_O_4_ formation. The authors of [9] presented a comparative analysis of mill scale reduction by carbon monoxide. Scale was in its original form and grounded to expand the surface and to account for the secondary oxidation in contact with air. Based on the thermal analysis data, it is shown that the process kinetics changes for scale of different fractions. The reduction process was investigated at temperatures of 850 °C, 950 °C and 1050 °C taking into account the secondary oxidation in contact with air at temperatures of 300 °C, 350 °C and 400 °C. The most favorable results were obtained for scale in the original form at T = 1050 °C. Most of the investigated iron oxide systems have a monophase composition. Data on the study of the MCA effect on iron-oxide systems of polyphase composition, such as scale, are few in the literature. Therefore, this paper presents the results when assessing the influence of two types of MCA (grinding and pressing) on the structure and properties of scale. Their influence on the reduction kinetics of scale iron oxides during heating has been evaluated.

Scale is a unique object of research because it contains basic iron oxides, arranged in layers according to the iron-oxygen state diagram. Iron oxides are represented by two-valent iron oxide-wustite (FeO), three-valent iron oxide-hematite (Fe_2_O_3_) and one-and-a-half oxide-magnetite (Fe_3_O_4_). Wustite [10] is a thermodynamically unstable compound, and below 560 °C it decays into α-Fe and magnetite (Fe_3_O_4_). The crystal lattice is cubic, a = 4.29 Å. The wustite crystal lattice always contains an excess of oxygen, therefore the composition of wustite more precisely corresponds to the non-stoichiometric formula Fe_0.91_O or FeO_1.09_. Due to this circumstance, the wustite crystal lattice contains a significant number of non-stoichiometry defects in the form of Fe^2+^ ion vacancies. From a structural point of view [11], wustite refers to solid subtraction solutions: in its lattice, oxygen atoms occupy all nodes in the anionic sublattice, and some nodes in the cationic sublattice are not occupied by iron atoms. With a lack of Fe^2+^ cations, lattice electroneutrality is maintained due to the partially transition of the two-charged Fe^2+^ cations to three-charged Fe^3+^. Therefore, this non-stoichiometric compound can be considered as a solid Fe_2_O_3_ substitution solution in FeO. The hematite (Fe_2_O_3_) is characterized by a rhombohedral lattice of the corundum type with a = 5.42 Å and α = 55°17’. The unit cell of hematite is formed by four Fe^3+^ and six O^2•^ ions. The latter form a dense hexagonal package. One-third of the octahedral voids are vacant, and two-thirds are filled with Fe^3+^. The magnetite (Fe_3_O_4_) has a cubic lattice of “reversed” spinel with a = 8.38 Å. The magnetite unit cell contains 8Fe^2+^, 16Fe^3+^ and 32O^2•^. Oxygen ions form the densest cubic lattice containing 32 octahedral and 64 tetrahedral voids in one unit cell. At the same time, eight Fe^2+^ ions and the same amount of Fe^3+^ are distributed (statistically) in octahedral, and the eight Fe^3+^ are distributed in tetrahedral interstices. The presence of dissimilar iron ions (Fe^2+^ and Fe^3+)^ in the same crystallographic positions facilitates the exchange of electrons and makes magnetite highly conductive.

In accordance with the principle of the sequence of transformations, iron oxide reduction during heating above 570 °C proceeds according to the generally recognized Equation (1), proposed by A.A. Baykov [12,13]:

(1) $${\mathrm{Fe}}_{2}O_{3}\overset{\left(C\left(\mathrm{CO}\right)\right)}{\to }{\mathrm{Fe}}_{3}O_{4}\overset{\left(C\left(\mathrm{CO}\right)\right)}{\to }\mathrm{FeO}\overset{\left(C\left(\mathrm{CO}\right)\right)}{\to }\mathrm{Fe}$$

To explain the effects observed during the scale iron oxide reduction, an ion-diffusion-catalytic mechanism of solid-phase technogenic (scale) iron oxide reduction was proposed in [5]. In accordance with this mechanism, at the first stage there is a solid-phase interaction of hematite located on the scale surface with solid carbon located in the intergranular space, with the formation of CO according to Equation (2):

Fe_2_O_3_ + C = 2FeO + CO↑ (2)

Thermodynamically, Equation (2) is possible at a temperature of 600 °C. Oxygen, interacting with carbon, in order to preserve the scale electroneutrality, transfers two electrons to neighboring iron atoms. As a result, an oxygen vacancy is formed on the scale surface simultaneously with two divalent iron atoms and, accordingly, two wustite molecules. 

The formed wustite molecules are thermodynamically unstable, since in the entire temperature range they are thermodynamically predisposed to interact with the layers of hematite located below. This leads to formation of two magnetite molecules by Equation (3):

2FeO + 2Fe_2_O_3_ = 2Fe_3_O_4_ (3)

The Gibbs free energy of this reaction is near 600 °C is −58.4 kJ/mol. At the second stage, the surface oxygen of the newly formed magnetite molecules will interact with solid intergranular carbon with the formation of 6 FeO and CO molecules according to Equation (4):

2Fe_3_O_4_ + 2C = 6FeO + 2CO↑ (4)

The Equation (4) is thermodynamically possible from 700 °C. As a result, oxygen will transfer electrons to neighboring iron ions in order to preserve electroneutrality, with the formation of six divalent iron ions (six wustite molecules, respectively) and two oxygen vacancies. As a reaction 4 result, newly formed wustite molecules will also associate with neighboring hematite molecules to form magnetite molecules. Due to the formation of a significant number of wustite molecules, the scale reduction processes will be catalytically accelerated with the divalent iron ions advancing deep into the scale, and oxygen ions transferring to the surface, towards oxygen vacancies.

At the final stage, only wustite molecules and oxygen vacancies will be located on the scale surface. The interaction of oxygen wustite with solid carbon will lead to the formation of metallic iron and CO according to Equation (5):

FeO + C = Fe + CO↑(5)

This reaction is thermodynamically possible from 800 °C. During the third stage, divalent iron and oxygen ions will diffuse to the surface, and metallic iron atoms will diffuse deep into the crystal. 

The proposed ion-diffusion-catalytic mechanism [5] makes it possible to describe effects observed during scale with carbon heating, but not the causes that triggered these effects. In [3], a scheme for preparing the raw mixture was used which differs from the classical scheme for preparing pellets. The raw mixture was crushed to a specific surface area by Blaine ≈ 400 m^2^/kg, followed by pressing at pressures of 100, 200 and 300 MPa. Since such preparation of raw materials for firing showed high efficiency, it was decided to evaluate the effect of two types of MCA (grinding and pressing) of the raw mixture on the reduction reaction kinetics. Changing the classical metallurgical scheme for preparing the raw mixture for firing by pelleting and for agglomeration for cold briquetting would significantly reduce the consumption of coke.

## 2. Materials and Methods

**Figure 1:**
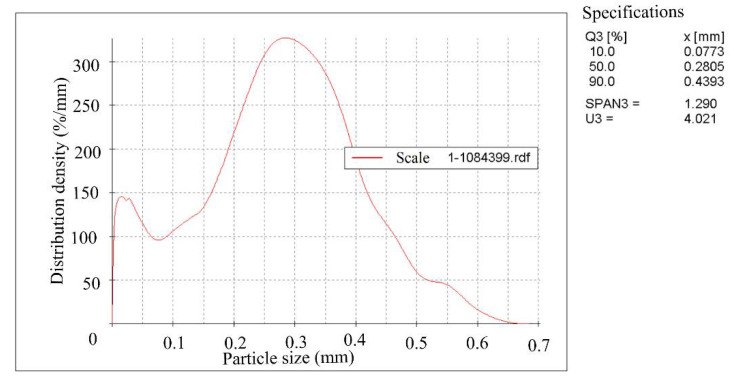
The scale granulometric composition, grounded to 1084 cm^2^/g.

**Figure 2:**
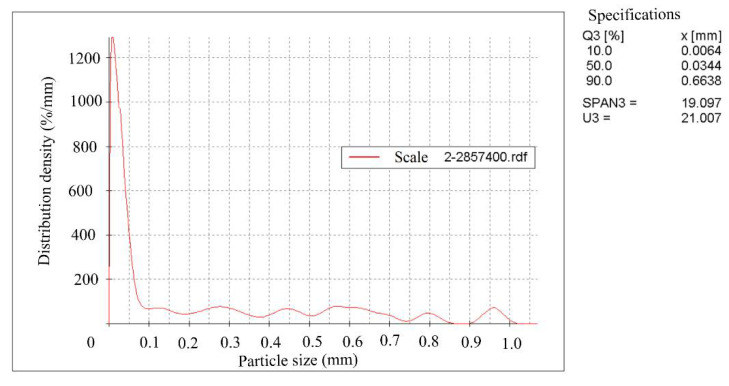
The scale granulometric composition, grounded to 2857 cm^2^/g.

**Figure 3:**
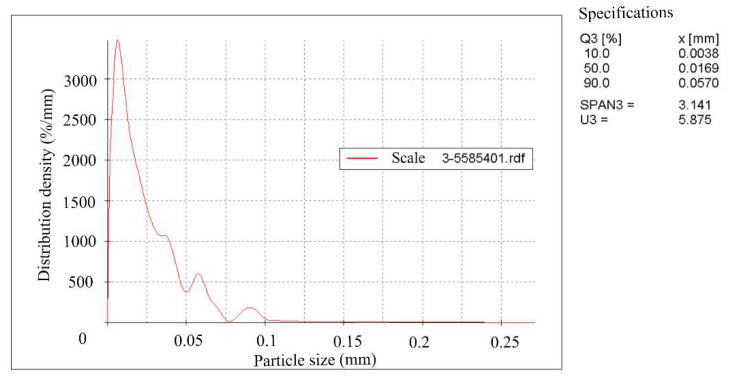
The scale granulometric composition, grounded to 5585 cm^2^/g.

Rolling scale was used during the work. The crystal phases’ identification and their quantitative ratios in the products were determined using X-ray phase a quantitative method of analysis on a diffractometer STADI-P (STOE, Darmstadt, Germany). The initial scale contains, according to quantitative X-ray phase analysis by mass %: 55.73 Fe_2_O_3_; 27.05 Fe_3_O_4_ and 17.22 FeO. The scale was dried to a constant mass and subjected to grinding in a bead mill with different grinding durations. The specific surface area of the grinding products was determined on the device for measuring the specific surface area and average particle size of powders (PSH-11M) and the grinding products’ granulometric composition was determined on the Camsizer XT device (RETSCH GmbH, Haan, Germany,). The specific surface area of the grinding products was 0 cm^2^/g, (assumed for the initial scale), 1084 cm^2^/g, 2857 cm^2^/g and 5585 cm^2^/g. The average particle size for 1084 cm^2^/g was 10.1 mkm, for 2857 cm^2^/g—3.8 mkm, for 5585 cm^2^/g—2.0 mkm. Figure 1, Figure 2 and Figure 3 show the scale granulometric composition, ground to a specific surface of 1084–2857–5585 cm^2^/g.

The dry ground scale of each specific surface was pressed without the introduction of any additives at pressures of 0 (initial scale), 100, 200 and 300 MPa.

The products of scale grinding and pressing were subjected to X-ray phase analysis to determine the phase composition.

After determining the phase composition, the second batch of raw mixture was prepared. The coke was added to the scale at an amount of 15% over stoichiometry. The samples of the second batch were crushed and pressed under the same conditions as the first and subjected to differential thermal analysis. Thermo-analysis was performed using the differential scanning calorimetry method (DSC) on the STA 449 F3 Jupiter thermo-analyzer (Netzsch-Geratebau GmbH, Selb, Germany). The experiment was conducted in an argon atmosphere (Ar high purity 99.998%, the volume fraction CO_2_ not more than 0.00002%), the gas flow was 30 mL/min under the condition of linear heating at a speed of 10 K/min, and the temperature measurement error was no more than ±1.5 degree. Al_2_O_3_ crucibles with lids were used for the study. Lids were holed to improve the reaction gas exchange. The value of the endothermic effect was calculated in the device software module in J/g and converted to kJ/mol by multiplying the device data by the mole mass of metallic iron. The firing products were subjected to quantitative X-ray phase analysis.

## 3. Results and Discussion

**Figure 4:**
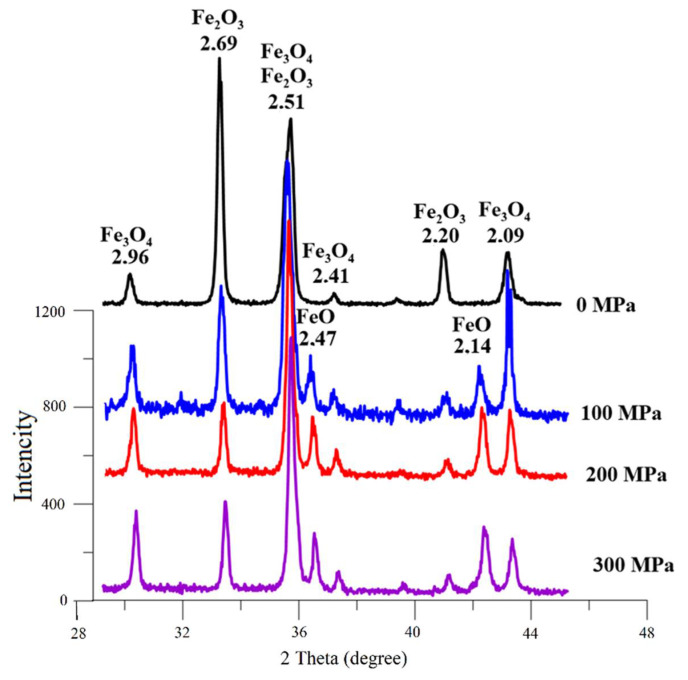
Influence of briquetting pressure at room temperature on the scale phase composition.

At the first stage of research, according to quantitative X-ray phase analysis, the separate effect of the briquetting pressure, the grinding degree on the scale phase composition at normal temperature and their combined effect were evaluated. The pressed samples phase composition change depending on the briquetting pressure according to the X-ray phase analysis data is shown in Figure 4 and in Table 1. 

A qualitative analysis of the briquetting pressure effect on the scale phase composition according to the X-ray phase analysis data (Figure 4) indicates that the non-pressed scale and scale pressed at 100 MPa completely lack diffraction peaks that are characteristic for FeO (interplane distances d = 2.14 Å and 2.47 Å). These peaks appear only at a briquetting pressure of 100 MPa and their intensity increases with increasing briquetting pressure. It can be noted that the intensity of diffraction peaks characteristic for Fe_3_O_4_ increases along with briquetting pressure increase according to Figure 4, and the diffraction peaks characteristic for Fe_2_O_3_ decrease in the same time.

**Figure 5:**
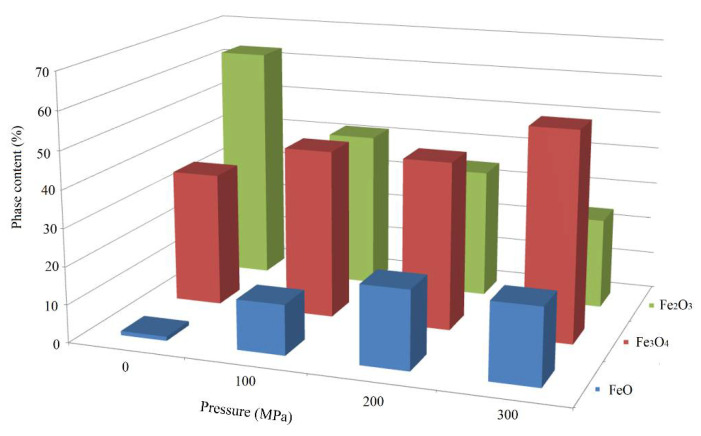
The nature of the scale phase composition change depending on the briquetting pressure.

The scale phase composition change pattern depending on the briquetting pressure is illustrated by Figure 5.

According to the data shown in Figure 5, as briquetting pressure increases, the content of Fe_2_O_3_ decreases, and Fe_3_O_4_ and FeO increases.

A quantitative assessment of the effect of briquetting pressure on the phase composition of the scale is given in Table 1.

Quantitative assessment of the briquetting pressing effect on the scale phase composition (Table 1) indicates that, with an increase in the scale briquetting pressure from 0 to 300 MPa, the Fe_2_O_3_ content decreases from 63.18 to 27.17%, the Fe_3_O_4_ content increases from 27.05 to 41.61%, and the FeO content increases from 17.22 to 30.22%.

**Figure 6:**
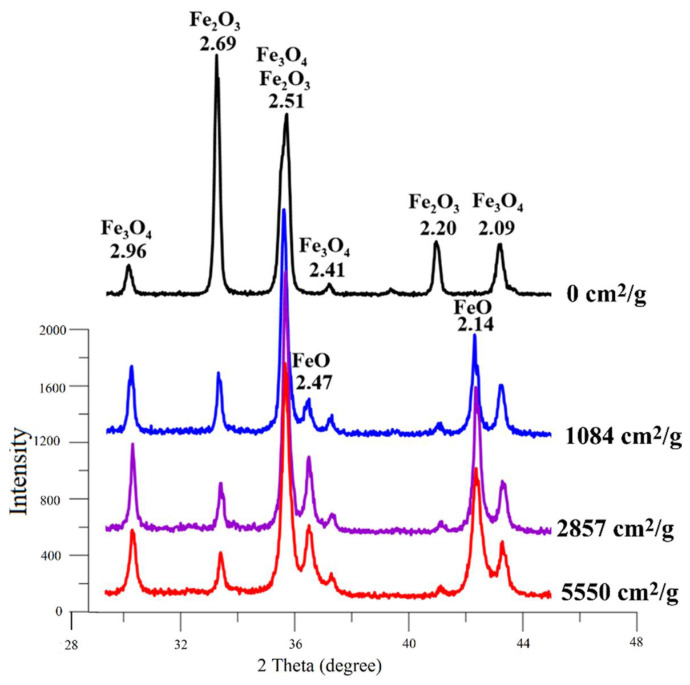
Influence of the grinding degree at room temperature on the scale phase composition.

A qualitative analysis of the grinding degree effect on the scale phase composition according to the X-ray phase analysis data (Figure 6) indicates that the non-ground scale completely lacks diffraction peaks that are characteristic of FeO (interplane distances d = 2.14 Å and 2.47 Å). These peaks appear only at a grinding degree of 1084 cm^2^/g and their intensity increases along with increased grinding degree. It can be noted that the intensity of the diffraction peaks characteristic of Fe_3_O_4_ increases slightly along with an increase in the grinding degree, and the diffraction maxima characteristic of Fe_2_O_3_ decreases at the same time.

**Figure 7:**
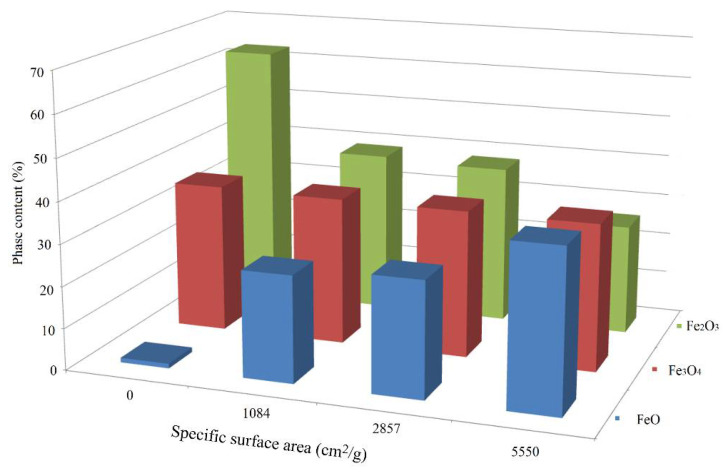
The nature of the scale phase composition change depending on the grinding degree.

The scale phase composition change pattern depending on the grinding degree is illustrated by Figure 7.

According to the data shown in Figure 7, along with an increase in the grinding degree, the content of Fe_2_O_3_ decreases, and Fe_3_O_4_ and FeO increases. It can also be noted that with an increase in the grinding degree up to 1084 cm^2^/g, the increase in FeO content occurs abruptly, and the increase in Fe_3_O_4_ over the entire grinding range is insignificant. 

A quantitative assessment of the grinding degree effect on the scale phase composition is given in Table 2.

A quantitative assessment of the grinding degree effect on the scale phase composition, given in Table 3, indicates that with an increase in the scale grinding degree from 0 cm^2^/g to 5550 cm^2^/g, the content of Fe_2_O_3_ decreases from 63.18 to 24.97%, the content of Fe_3_O_4_ increases from 27.05 to 46.93%, and the content of FeO increases from 17.22 to 28.1%.

The combined results of pressing and grinding on the scale phase composition at room temperature according to phase analysis are shown in Table 3.

**Figure 8:**
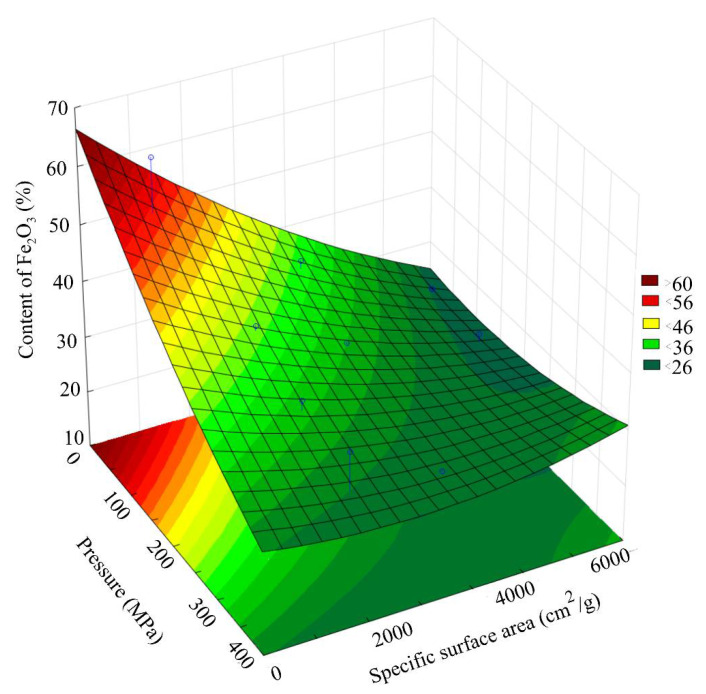
Response function of the Fe_2_O_3_ content in the scale depending on the combined effect of the briquetting pressure and the grinding degree.

**Figure 9:**
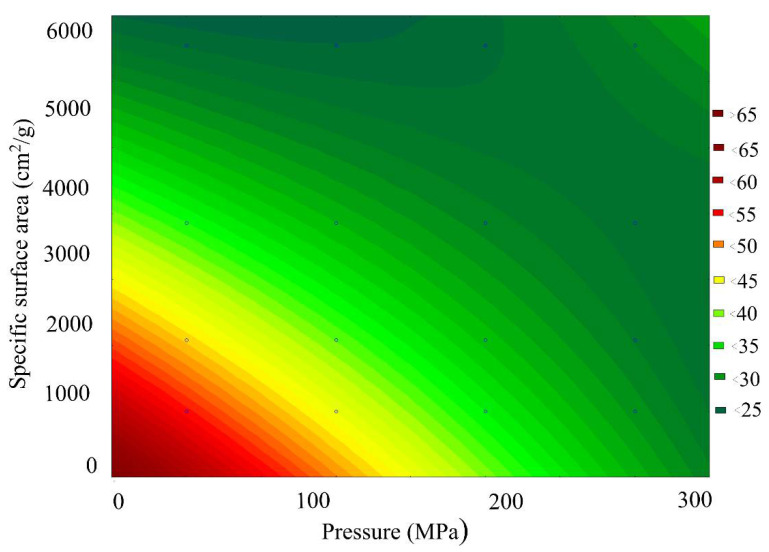
Isolines of equal Fe_2_O_3_ content in scale, depending on the combined effect of briquetting pressure and the grinding degree.

Figure 8 presents a general view of the response function of the Fe_2_O_3_ content in the scale, depending on the combined effect of the briquetting pressure and the grinding degree at normal temperature. Figure 9 shows isolines equal to Fe_2_O_3_ content for this function.

Analysis of the Fe_2_O_3_ content changes pattern, depending on the grinding and pressing combined effect, indicates that under the isoline corresponding to the grinding degree of 3000 cm^2^/g and the briquetting pressure of 200 MPa, there is a sharp decrease in the amount of Fe_2_O_3_ by 25–30%. With a further increase in the MCA intensity, the content of Fe_2_O_3_ changes slightly. 

**Figure 10:**
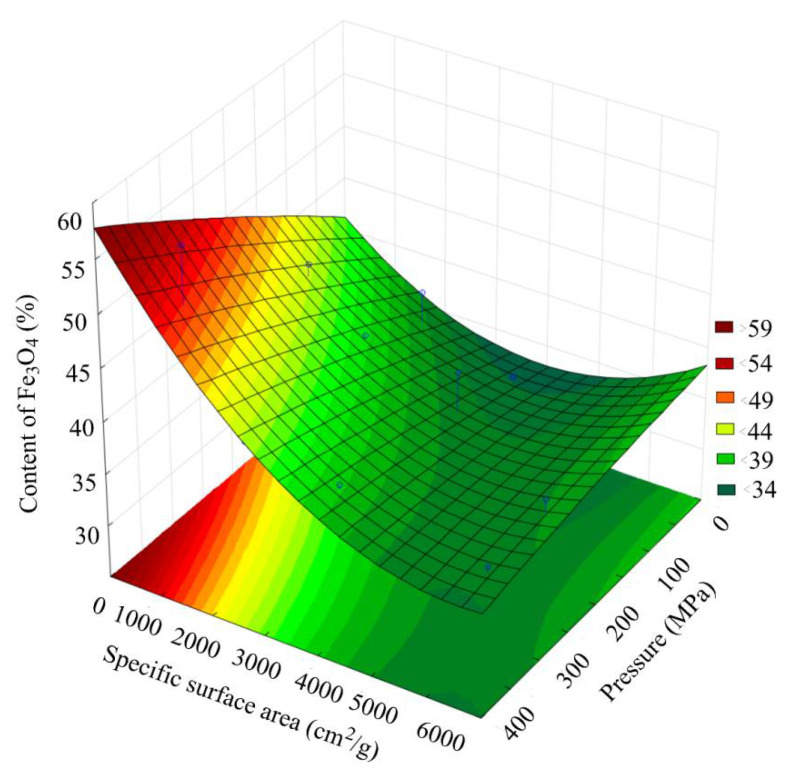
Response function of Fe_3_O_4_ content in scale depending on the combined effect of briquetting pressure and the grinding degree.

**Figure 11:**
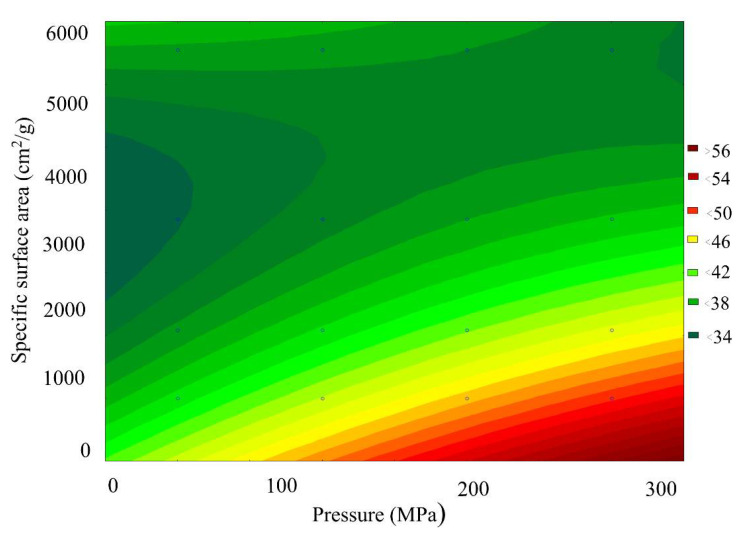
Isolines equal the content of Fe_3_O_4_ in the scale, depending on the combined effect of briquetting pressure and the grinding degree.

Figure 10 presents a general view of the response function of the Fe_3_O_4_ content in the scale, depending on the combined effect of the briquetting pressure and the grinding degree. Figure 11 shows that isolines equal Fe_3_O_4_ content for this function.

Analysis of the Fe_3_O_4_ content changes pattern, depending on the grinding and pressing combined effect, indicates that along with an increase in the briquetting pressure, the content of Fe_3_O_4_ increases, and decreases along with an increase in the grinding degree.

**Figure 12:**
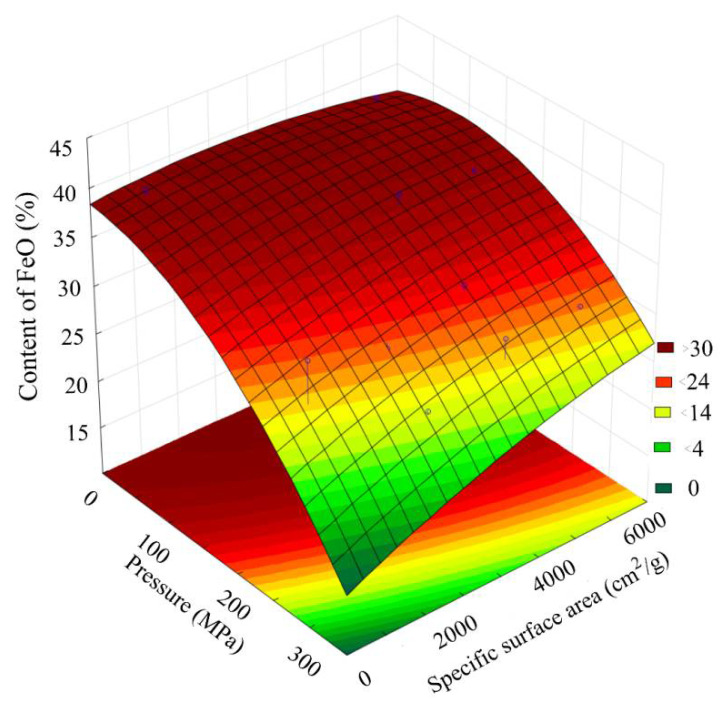
The response function of the FeO content in the scale depending on the combined effect of the briquetting pressure and the grinding degree.

**Figure 13:**
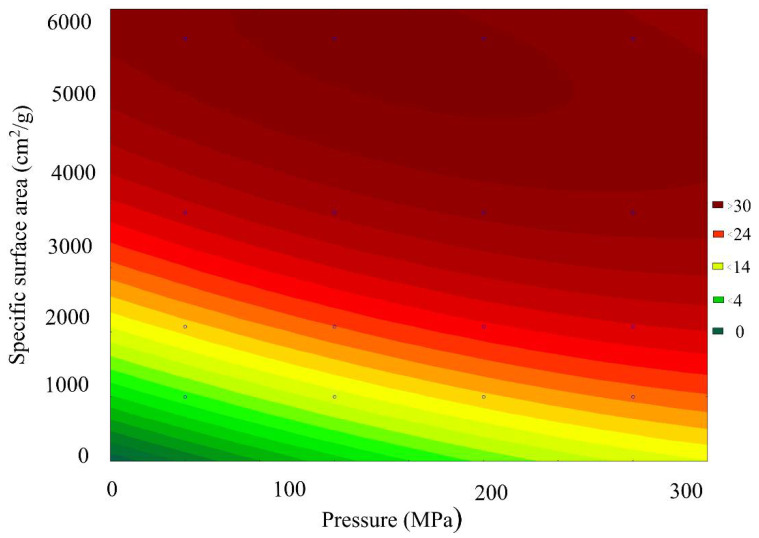
Isolines of equal FeO content in scale, depending on the combined effect of briquetting pressure and the grinding degree.

Figure 12 presents a general view of the response function of the FeO content in the scale, depending on the combined effect of the briquetting pressure and the grinding degree. Figure 13 shows that isolines equal FeO content for this function.

Analysis of the FeO content changes pattern, depending on the grinding and pressing combined effect, indicates that at a briquetting pressure of 150 MPa and a grinding degree of 5550 cm^2^/g, an optimal area with a maximum FeO content is observed.

During the experiment planning to assess the MCA effect on scale reduction, it was assumed that under mechanical impact, scale crystal structure would amorphized, as described in the above literature sources, and the amorphization degree could be determined by reducing the intensity and increasing the half-width of the diffraction maxima of scale iron oxide phases. However, the analysis of the experimental results showed that there is no scale crystal structure amorphization as a result of MCA, but a change in its phase composition, spotted in a decrease in the Fe_2_O_3_ content and an increase in the content of Fe_3_O_4_ and FeO. Descriptions of such an effect could not be found in the literary sources. The change in the phase composition of iron oxides during MCA is described in the work of [8]; however, this was spotted in the presence of graphite, which is theoretically understandable. The necessary and sufficient thermodynamic conditions for their reaction can be achieved during prolonged joint mechanical activation of iron oxide and graphite. Under the conditions of our experiment, carbon was not introduced into the scale in any form, so it is impossible to explain the observed effect according to classical ideas about the phase transformations of iron oxide in the presence of carbon.

**Figure 14:**
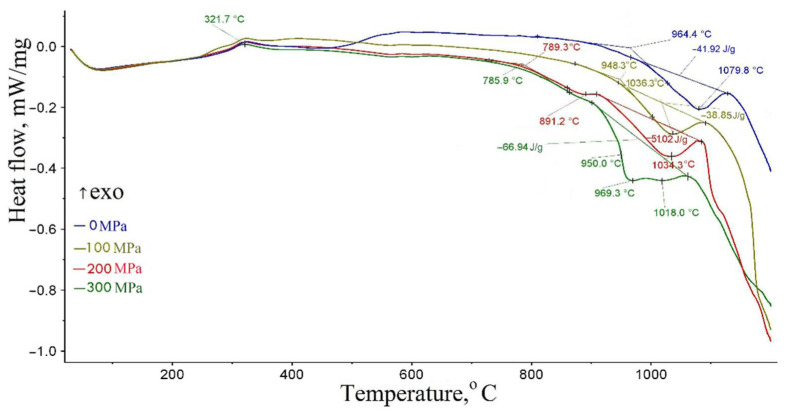
The DSC data of samples pressed with coke.

**Figure 15:**
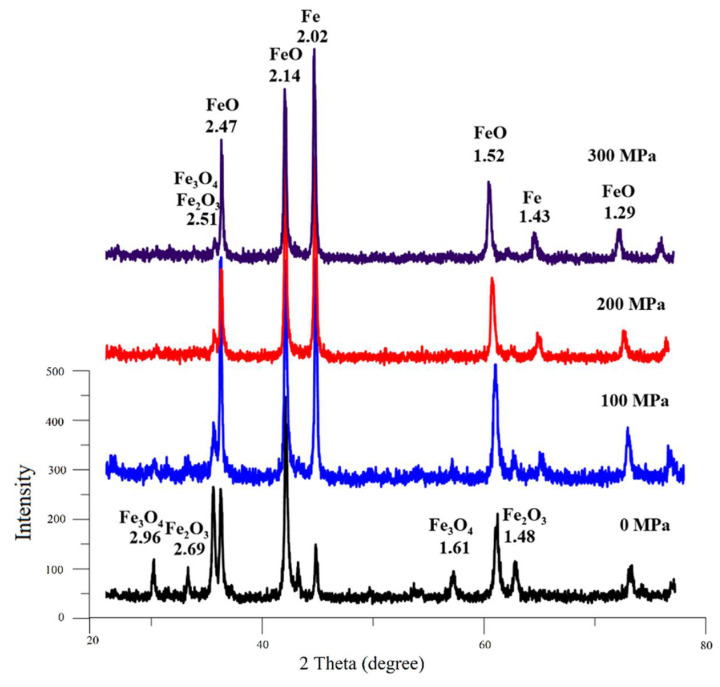
The phase composition changes of calcined samples pressed with coke.

At the second stage of the research, the MCA effect at normal temperature on the process of reducing scale with coke during their calcination was determined. The DSC data of samples pressed with coke are presented in Figure 14. The phase composition of calcined samples pressed with coke is shown in Table 4. The phase composition changes of the calcined samples pressed with coke according to the X-ray phase analysis is shown in Figure 15. 

An analysis of the results presented in Figure 14 and Figure 15 and in Table 4 indicates that they confirm the results described in [5]. 

With an increase in the briquetting pressure (Figure 14), the beginning of metallization decreases from 964.4 at 0 to 789.5 °C at 300 MPa, and the enthalpy of metallization increases from 41.92 at 0 to 66.94 J/g at 300 MPa. These effects in [5] are explained by an increase in the defect of the pressed samples.

On the diffractogram of non-pressed scale (Figure 15), the residues of Fe_2_O_3_ and Fe_3_O_4_ are spotted. These phases are not observed on pressed samples. This effect can also be explained by an increase in the defectiveness of the pressed samples’ crystal lattice, since it increases the reduction reaction rate. 

**Figure 16:**
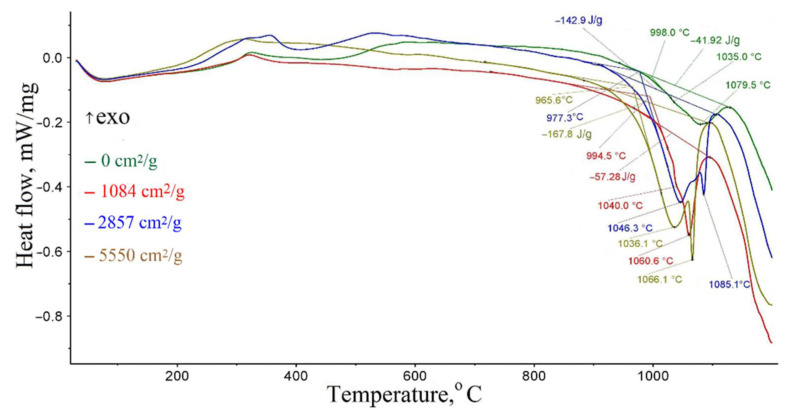
The DSC data of samples ground with coke.

**Figure 17:**
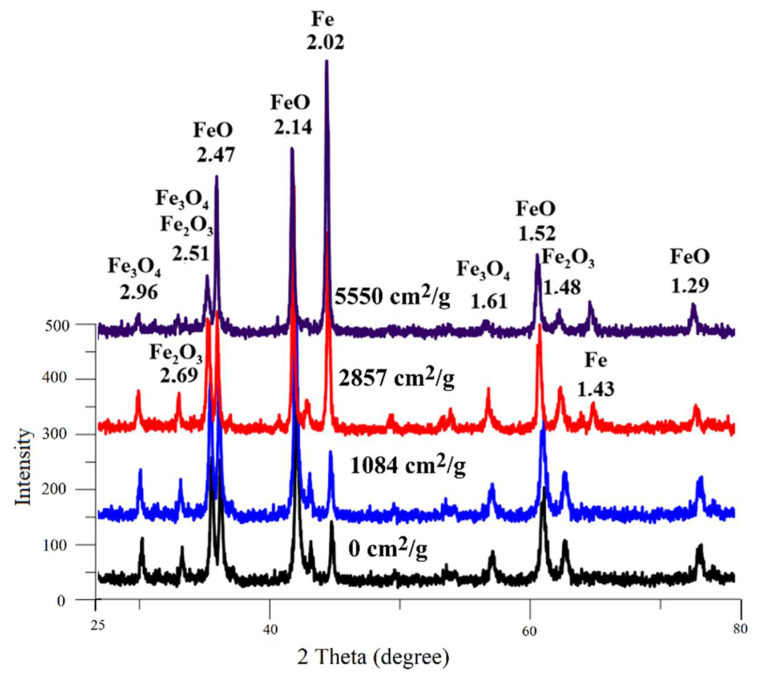
The change in the phase composition of calcined samples ground with coke.

**Figure 18:**
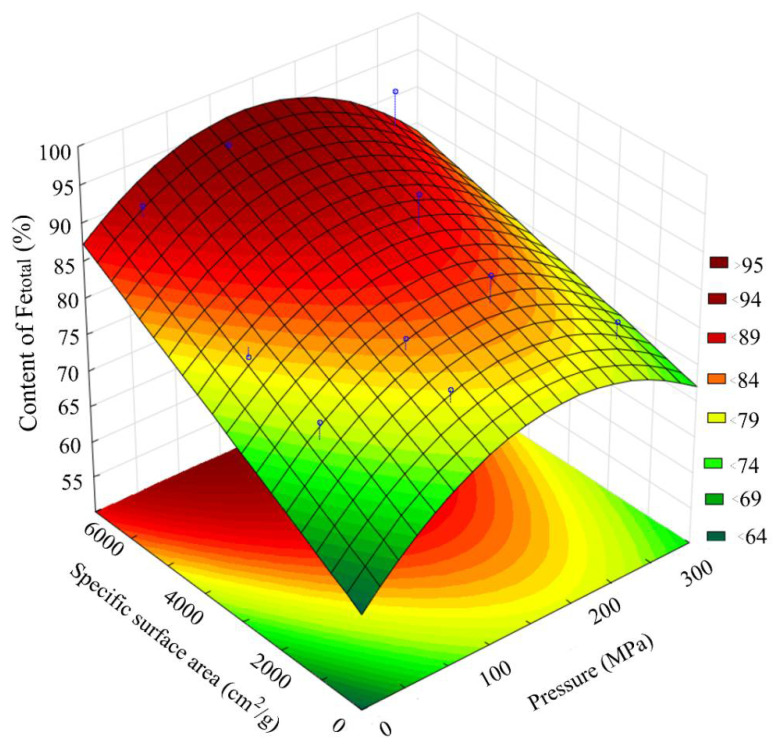
The effect of joint MCA on the total iron content in calcined samples.

**Figure 19:**
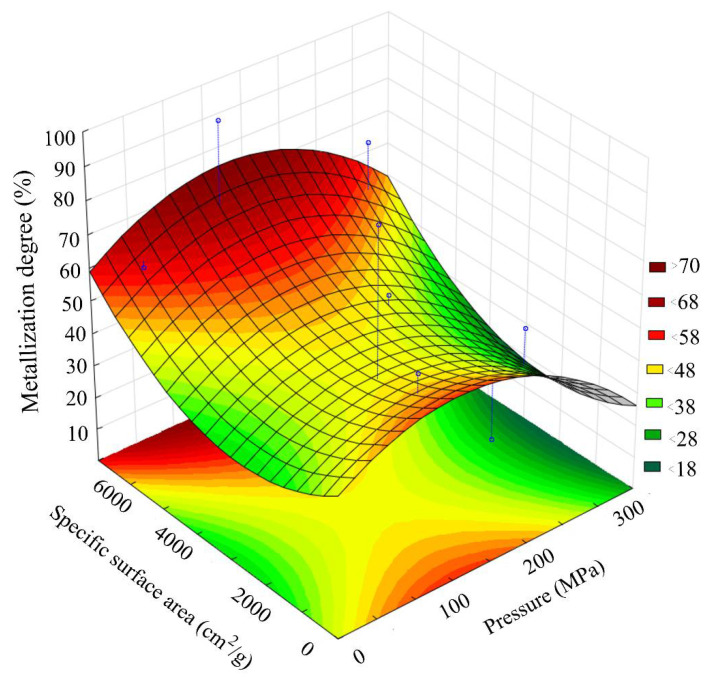
The effect of joint MCA on the calcined samples’ metallization degree.

The DSC data of samples ground with coke are shown in Figure 16. The phase composition of calcined samples ground with coke is shown in Table 5. The change in the phase composition of calcined samples ground with coke is shown in Figure 17. Table 6 shows data on the effect of joint MCA on the metallization degree of calcined samples. Figure 18 shows the effect of joint MCA on the total iron content in calcined samples. Figure 19 shows the effect of joint MCA on the calcined samples’ metallization degree.

Figure 16 and Figure 17 and the results analysis in Table 5 show that with an increase in the grinding degree (Figure 16), the metallization temperature shifts down from 998.0 at 0 to 965.6 °C at 5550 cm^2^/g, and the value of the metallization enthalpy increases from 41.92 at 0 to 167.8 J/g at 5550 cm^2^/g. These effects can also be explained by an increase in the defectiveness of grounded samples, and according to the enthalpy value, it can be noted that the crystal lattice defectiveness during grinding is about three times higher than during pressing.

The results of Fe_total_ and metallization degree determination shown in Figure 18 and Figure 19 correlate well with the data on the FeO content shown in Figure 12 and Figure 13. The maximum metallization degree and the Fe_total_ content are in the region with a briquetting pressure of 150 MPa and a grinding degree of 5550 cm^2^/g. We supposed that the largest number of crystal lattice defects accumulates in this region, which make it easier to reduce FeO to Femet.

The test results indicate that MCA leads to a scale phase composition change both by scale pressing and by grinding at normal temperature, without carbon participation. This all corresponds to the initial stages of iron oxide reduction in accordance with the A.A. Baykov Equation (1). It can be noted that the scale phase transformations during the observed effects occur in accordance with Equation (6):

(6) $${\mathrm{Fe}}_{2}O_{3}\overset{\;Em}{\to }{\mathrm{Fe}}_{3}O_{4}\overset{Em\;}{\to }\mathrm{FeO}$$

where *Em* is MCA energy, obtained by scale during mechanical treatment, in kJ/mol.

The energy of the system changes under the MCA effect. Our conclusion is confirmed by the authors of [14]. They report that the activation of chemical processes is associated with the release of elastic energy at the solid destruction moment, the rupture of chemical bonds and the formation of short-lived active centers (radicals with uncompensated valence). It is considered in dislocation theory that activation occurs due to the energy of dislocations coming to the surface during plastic crushed substance deformation. Plastic deformation (both pressing and grinding) leads to various defects. Structural defects occur in solids under the influence of shear stresses. This leads to the substance dissociation.

The main process that MCA provides during the scale phase composition is an iron valence change from Fe^3+^ to Fe^2+^. Based on the data presented in Table 1 and Table 2, with a decrease in the content of Fe_2_O_3_ by 40%, there is an increase in the content of Fe_3_O_4_ by 20% and FeO by 20%. Such a change in the phase composition can be described by Equation (7) or (8):

(7) $$2{\mathrm{Fe}}_{2}O_{3}\;\overset{Em}{\Rightarrow }{\mathrm{Fe}}_{3}O_{4}\;+\;\mathrm{FeO}\;+\;O\left(g\right)$$

(8) $$4{\mathrm{Fe}}_{2}O_{3}\overset{Em}{\Rightarrow }2{\mathrm{Fe}}_{3}O_{4}+2\mathrm{FeO}\;+\;O2\left(g\right)$$

We assume, that the energy obtained by the scale under mechanical treatment contributes to the electronic orbitals’ displacement of oxygen and iron ions. As a result, two ions Fe^3+^ of hematite capture two electrons of oxygen, becoming two Fe^2+^ ions. The oxygen ion O^2•^ becomes electroneutral and diffuses to the surface of the scale. Due to the change in the valence of two hematite iron ions and the release of one oxygen atom from the hematite molecule, two FeO molecules are formed. One of them is immediately associated with the Fe_2_O_3_ molecule via the Fe_3_O_4_ formation in Equation (3). The Gibbs free energy of this reaction is −25.575 kJ/mol at room temperature. As a result of Equations (8) and (9), two moles of hematite are converted into one mole of wustite and one mole of magnetite, which corresponds to the scale phase content change observed during experiment.

Equation (7) is thermodynamically possible from a temperature of 2200 °C, and Equation (8) from a temperature of 1700 °C. It is obvious that the amount of thermal energy required for Equation (7) should be equal to the amount of mechanical energy obtained by the scale under mechanical treatment:

*Et* = *Em* (9)

where, *Et*—thermal energy, kJ/mol.

Equation (9) could be presented as 9 for the pressing pressure:

C · dT · M = P · dV + Ed(10)

where c—scale specific heat capacity, J/g·deg;

dT— Equation (7) realization temperature, K; 

M—hematite molar mass, mol; 

P—briquetting pressure, MPa; 

dV—scale deformation during pressing, sm^3^; 

Ed—total energy of scale defects formed during pressing or grinding, J/mole.

The measurement of the true density of the pressed samples by the pycnometric method showed that at briquetting pressures above 200 MPa, the porosity of the samples tends to 0 and the average density to the true density. Measuring of pressed samples deformation at a pressure of 300 MPa show that the system obtained 7.59 kJ/mol of mechanical energy during pressing, while the required thermal energy for Equation (7) is 882.11 kJ/mol. The difference is approximately 100 times. We consider that the greatest energy contribution to the process is made by the energy of defects in the system (Ed). Change of that energy can be estimated only by indirect methods.

According to Boldyrev [15,16,17,18], defects are concentrators of stress and excess energy of the crystal lattice. To anneal these defects, it is necessary to spend additional energy. This should be reflected in the scale metallization enthalpy value. The DSC data analysis of the samples pressed and ground with coke, shown in Figure 14 and Figure 16, indicates that with an increase in the briquetting pressure and the degree of scale grinding, the metallization enthalpy value really increases (Table 7). These data also show that with an increase in the briquetting pressure and degree of grinding, the beginning of metallization shifts to the low temperatures.

With an increase in the briquetting pressure and grinding degree, the enthalpy of metallization increases. This indicates that with MCA increase, the iron oxide crystal lattice defectiveness increases. Additional energy is required to anneal these defects. An increase in the iron oxide’s crystal lattices defectiveness also facilitates the diffusion processes. This leads to a shift in the metallization temperature. 

The DSC data (Figure 16) shows a double endothermic effect at grinding degrees to a specific surface of 2857 and 5550 cm^2^/g. The appearance of a double endothermic effect can be explained by the presence of a significant amount of magnetite in the samples after grinding at normal temperature. The residual amount of Fe_3_O_4_ is revealed after annealing (Figure 15). The first peak of the endothermic effect corresponds to the Fe_3_O_4_ recovery, and the second to the FeO recovery.

## 4. Conclusions

1. It has been experimentally established that the mechanical activation (grinding: 1084; 2857; 5550 cm^2^/g; or pressing 100; 200; 300 MPa) have a significant effect on the kinetics of iron oxide reduction reactions.
2. The scale MCA at room temperature without reducing agent activates the iron oxide reduction process. This occurs due to a change in the energy of the system during MCA and is associated with the rupture of chemical bonds and scale defects formation. It reveals a decrease in the amount of trivalent iron oxide (Fe_2_O_3_) and an increase in the amount of one-and-a-half oxide (Fe_3_O_4_) and ferrous iron oxide (FeO). The quantitative change of phases both during pressing and grinding under the conditions of the experiment is approximately the same. The observed effects are associated with the change in the iron valence from Fe^3+^ to Fe^2+^ after mechanical treatment.
3. According to XRD data, the scale phase content change patterns differ with different MCA types. The change in the specific surface area of the grinded products to 1084 cm^2^/g leads to an abrupt FeO amount increase. Further increase in the grinding degree increases the content of FeO and Fe_3_O_4_ phases slightly. Increasing the briquetting pressure monotonically reduces the amount of Fe_2_O_3_ and increases the amount of Fe_3_O_4_ and FeO.
4. The scale phase composition change under the MCA effect leads to a change in the kinetics of the scale reduction reactions. The DSC curves course showed that an increase in the briquetting pressure and the grinding degree leads to an increase in the scale metallization process enthalpy and a decrease in its temperature.
5. The change in the scale metallization kinetic parameters (the metallization temperature shifting down to low temperatures and the metallization rate increasing) after the MCA is the consequence of the elimination of crystal lattice defects in scale iron oxides.

## Figures and Tables

**Table 1:** Effect of briquetting pressure at room temperature on the phase composition of scale.

Briquetting Pressure, MPa	Content, Mass. %		
	Fe_2_O_3_	Fe_3_O_4_	FeO
0	63.18	27.05	17.22
100	49.99	35.84	14.17
200	36.5	41.91	21.58
300	27.17	41.61	30.22

**Table 2:** Influence of the grinding degree at normal temperature on the scale phase composition.

Specific Surface of Grounded Scale, cm^2^/g	Content, Mass. %		
	Fe_2_O_3_	Fe_3_O_4_	FeO
0	63.18	27.05	17.22
1084	24.47	48.94	26.6
2857	20.83	45.28	33.89
5550	24.97	46.93	28.10

**Table 3:** The scale phases content depending on the combined effect of briquetting pressure and grinding at normal temperature.

Briquetting Pressure, MPa	Specific Surface of Grounded Scale, cm^2^/g	Content, Mass. %		
		Fe_2_O_3_	Fe_3_O_4_	FeO
0	0	63.18	27.05	17.22
100	0	49.99	35.84	14.17
200	0	36.50	41.91	21.58
300	0	27.17	41.61	30.22
0	1084	24.47	48.94	26.6
100	1084	39.49	39.86	20.66
200	1084	35.08	38.50	26.42
300	1084	35.15	39.39	25.46
0	2857	20.83	45.28	33.89
100	2857	31.96	39.29	28.71
200	2857	28.04	35.47	36.54
300	2857	26.78	38.49	34.73
0	5550	24.97	46.93	28.10
100	5550	26.50	35.94	37.54
200	5550	25.47	36.96	37.72
300	5550	26.15	35.84	38.01

**Table 4:** The phase composition of calcined samples pressed with coke.

Briquetting Pressure, MPa	Content, Mass. %			
	Fe_2_O_3_	Fe_3_O_4_	FeO	Fe
0	14.72	35.95	44.42	4.90
100	7.00	8.80	62.70	21.48
200	2.86	3.81	61.89	31.43
300	3.29	1.84	61.25	33.60

**Table 5:** The phase composition of calcined samples ground with coke.

Specific Surface of Grounded Scale, cm^2^/g	Content, Mass. %			
	Fe_2_O_3_	Fe_3_O_4_	FeO	Fe
0	14.72	35.95	44.42	4.90
1084	15.55	29.87	34.69	19.89
2857	14.22	21.6	45.24	18.939
5550	0.60	1.23	74.31	23.852

**Table 6:** The effect of joint MCA on the metallization degree of calcined samples.

Briquetting Pressure, MPa	Specific Surface of Grounded Scale, cm^2^/g	Fe_all_, %	Metallization Degree, %
0	0	68.95	34.4
100	0	81.13	55.46
200	0	77.00	24.7
300	0	79.80	32.10
0	1084	78.12	36.48
100	1084	83.98	91.18
200	1084	87.35	31.50
300	1084	77.96	40.40
0	2857	80.40	35.94
100	2857	85.23	28.63
200	2857	92.18	48.30
300	2857	78.08	23.40
0	5550	91.40	58.37
100	5550	94.82	92.70
200	5550	88.52	36.00
300	5550	92.99	67.60

**Table 7:** The briquetting pressure and grinding degree effect on metallization temperature and its enthalpy.

Briquetting Pressure, MPa	Grinding Degree, cm^2^/g	Metallization Temperature, °C.	Metallization Enthalpy, kJ/mole
0	0	964.4	2.34
100	0	948.4	2.18
200	0	891.2	2.86
300	0	785.9	3.75
0	0	964.4	2.34
0	1084	994.5	2.93
0	2857	977.3	8.00
0	5550	965.6	9.39

## References

[1] Nasser A., Mingelgrin U. (2012). Mechanochemistry: A review of surface reactions and environmental applications. Appl. Clay Sci..

[2] Mondal K., Lorethova H., Hippo E., Wiltowski T., Lalvani S. (2004). Reduction of iron oxide in carbon monoxide atmosphere—Reaction controlled kinetics. Fuel Processing Technol..

[3] Drobíková K., Valová S., Motyka O., Kutláková K.M., Plachá D., Seidlerová J. (2018). Effects of binder choice in converter and blast furnace sludge briquette preparation: Environmental and practical implications. J. Waste Manag..

[4] Suarez S.M.E., Borja-Castro L.E., Valerio-Cuadros M.I., Domínguez A.B., Cabrera-Tinoco H.A., Huaman E., Valencia-Bedregal R.A., Zhao X., Zhang Y., Zhang D. (2021). Carbothermal reduction of mill scales formed on steel billets duing continuous casting. Hyperfine Interact..

[5] Sheshukov O.Y., Mikheenkov M.A., Vedmid L.B., Nekrasov I., Egiazaryan D. (2020). Mechanism of ion-diffusion solid-phase reduction of iron oxides of technogenic origin in the presence of the liquid phase and without it. Metals.

[6] Emelyanov D.A., Korolev K.G., Mikhailenko M.A., Knot’ko A.V., Oleinikov N.N., Tret’yakov Y.D., Boldyrev V.V. (2004). Mechanochemical synthesis of wustite in high-power apparatuses. J. Inorg. Mater..

[7] Ilyin A.A., Smirnov N.N., Ilyin A.P. (2005). Influence of mechanical activation on the structure and catalytic properties of iron oxide. J. Chem. Chem. Technol..

[8] Kozhina G., Estemirova S., Pechishcheva N., Murzakaev A., Vovkotrub E., Skrylnik M., Shunyaev K. (2017). Joint mechanical activation of MnO_2_, Fe_2_O_3_ and graphite: Mutual influence on the structure. Adv. Powder Technol..

[9] Nowacki K., Maciąg T., Lis T. (2021). Recovery of Iron from Mill Scale by Reduction with Carbon Monoxide. Minerals.

[10] Gerasimenko A.A., Aleksandrov Y.I., Andreev I.N., Batalov A.K., Beloglazov S.M., Bogoyavlenskiy A.F., Valeev N.N., Gerasimova V.V., Gerasimov V.V., Gonik A.A. (1987). Protection against Corrosion, Aging and Bio-Damage of Machinery, Equipment and Structures: Handbook in 2 Volumes.

[11] Gorshkov V.S., Savelyev V.G., Fedorov N.F. (1988). Physical Chemistry of Silicates and Other Refractory Compounds: Textbook.

[12] Baikov A.A. (1948). Collection of Selected Works in 2 Books Book 2.

[13] Sun G., Li B., Guo H., Yang W., Li S., Guo J. (2020). Thermodynamic study on reduction of iron oxides by H_2_ + CO + CH_4_ + N_2_ mixture at 900 °C. Energies.

[14] Avvakumov E.G., Gusev A.A. (2009). Mechanical Activation Methods in the Processing of Natural and Man-Made Raw Materials.

[15] Boldyrev V.V. (2006). Mechanochemistry and mechanical activation of solid. Russ. Chem. Rev..

[16] Boldyrev V.V. (1983). Experimental Methods in Mechanochemistry of Solid Inorganic Substances.

[17] Boldyrev V.V. (1979). Control of the reactivity of solids. Ann. Rev. Mater. Sci..

[18] Boldyrev V.V., Bulens M., Delmon B. (1979). The Control of Rectivity of Solids.

